# Electrospinning Technique for Fabrication of Coaxial Nanofibers of Semiconductive Polymers

**DOI:** 10.3390/polym14235073

**Published:** 2022-11-22

**Authors:** William Serrano-Garcia, Seeram Ramakrishna, Sylvia W. Thomas

**Affiliations:** 1 Advanced Materials Bio & Integration Research (AMBIR) Laboratory, Department of Electrical Engineering, University of South Florida, Tampa, FL 33620, USA; 2 Department of Mechanical Engineering, National University of Singapore, Singapore 117574, Singapore

**Keywords:** P3HT, BBL, organic semiconductors, flexible electronics

## Abstract

In this work, the electrospinning technique is used to fabricate a polymer-polymer coaxial structure nanofiber from the p-type regioregular polymer poly(3-hexylthiophene-2,5-diyl) (P3HT) and the n-type conjugated ladder polymer poly(benzimidazobenzophenanthroline) (BBL) of orthogonal solvents. Generally, the fabrication of polymeric coaxial nanostructures tends to be troublesome. Using the electrospinning technique, P3HT was successfully used as the core, and the BBL as the shell, thus conceptually forming a p-n junction that is cylindrical in form with diameters in a range from 280 nm to 2.8 µm. The UV–VIS of P3HT/PS blend solution showed no evidence of separation or precipitation, while the combined solutions of P3HT/PS and BBL were heterogeneous. TEM images show a well-formed coaxial structure that is normally not expected due to rapid reaction and solidification when mixed in vials in response to orthogonal solubility. For this reason, extruding it by using electrostatic forces promoted a quick elongation of the polymers while forming a concise interface. Single nanofiber electrical characterization demonstrated the conductivity of the coaxial surface of ~1.4 × 10^−4^ S/m. Furthermore, electrospinning has proven to be a viable method for the fabrication of pure semiconducting coaxial nanofibers that can lead to the desired fabrication of fiber-based electronic devices.

## 1. Introduction

Intelligent textiles, air/water filters, bone scaffolds, non-woven films, and drug delivery applications have all directly benefitted from the development of the reliable and low-cost electrospinning techniques for fiber fabrication [1,2,3,4,5,6]. This technique has also been successfully used to fabricate fibrous electronic devices, such as diodes, field effect transistors, photovoltaics, and sensors that use organic conductive-semiconductive polymers (C-SPs) and other relevant materials forming composites [6,7,8]. These materials are known to have preferred thermal stability [9], electrical conductivity [10,11], mechanical flexibility [12,13], and chemical/biological functionality [14,15]. Furthermore, polymeric materials have been researched in coaxial arrangements by electrospinning for use in drug delivery applications and the fabrication of hollow fiber channels, to name a few [16,17]. Previous work has also been reported on organic semiconducting p-n junction nanofibers in a coaxial core-shell (core-sheath) structure but with a focus on small-molecule (organic-organic) heterojunctions and partial organic (organic/polymer-inorganic) heterojunctions rather than polymeric heterojunctions [18]; however, to the best of our knowledge, electroactive polymer-polymer coaxial heterojunctions have not yet been fabricated [15]. Therefore, the fabrication of semiconducting-based coaxial nanofibers by using the electrospinning technique is the main task of this work.

Based on these previous works, we expect that organic semiconductive coaxial nanofibers could produce p-n junctions with electronic properties and fabrication reliability, thus making them excellent candidates for use in flexible electronics and cost-effective nanomanufacturing [12]. Because of this, we propose the electrospinning technique for the fabrication of an electroactive polymer-polymer heterojunction. More specifically, these novel organic semiconductive p-n junctions have the potential to demonstrate high I_on_/I_off_ ratios and, in field-effect transistor (FET) mode, a reduction in leakage current compared to thin film structures [19]. In diode mode, a tunable depletion region may be achieved by the phase created by the outer covering. In photovoltaic applications, the coaxial structure creates an optimal environment for the generation of charges derived from light irradiation, increasing the photocurrent in the semiconductor. Therefore, due to the large surface area that can be generated by nanofibers (non-woven mesh or aligned nanofibers) in a small region, a coaxial p-n junction matrix will be capable to perform as a sensor, photodiode, and as a photovoltaic cell. As a result, the surface of the nanofibers can be functionalized for specific sensing and higher selectivity, improving the performance for electronic applications [20].

Nanofibers of the semiconductive regioregular polymer poly(3-hexylthiophene-2,5-diyl) (P3HT, with field effect mobilities as high as 0.1 cm^2^V^−1^s^−1^) [21,22] and thin films, as well as self-assembled nanobelts, of the ladder polymer poly(benzimidazobenzophenanthroline) (BBL, with electron mobilities of approximately 1 × 10^−3^ cm^2^ V^−1^ s^−1^ and 7 × 10^−3^ cm^2^ V^−1^ s^−1^, respectively) [23,24] have been previously characterized as having morphologies and high-mobility behaviors that make them ideal candidates for organic electronics. [23,25,26]. However, fiber fabrication of pure P3HT is challenging (due to its low molecular weight [27]), and under laboratory conditions, this material tends to degrade over time due to oxygen exposure [28]. BBL, on the other hand, has been shown to exhibit high stability in air over a long period of time, thus improving the shelf life of the formed devices [29]. Polystyrene (PS) can be used to facilitate the formation of P3HT fibers.

For these reasons, the utility of organic semiconductive polymers-specifically, P3HT and BBL-in forming one-dimensional (1D) coaxial p-n junctions can lead to the fabrication of fully functional single-fiber organic electronics. The expectation is that devices can directly be tethered in a single functional fiber that may increase the efficiency and even lower power requirements for textile applications. More broadly, this research seeks to address the technological challenges of using nanoelectronics and nanosensors for flexible, low-power nanodevices (e.g., tethered and 1D components for chemical, vapor, and gas sensing) by enhancing the nanometric morphology of the coaxial structure [30,31].

## 2. Materials and Methods

**Figure 1:**
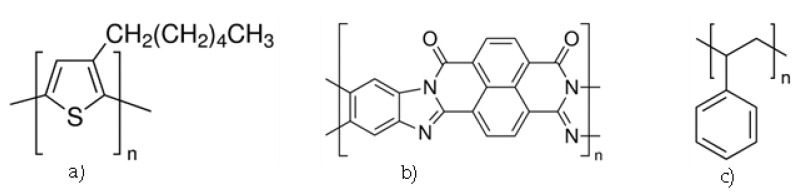
Coaxial nanofiber was fabricated by using P3HT (**a**) and BBL (**b**) as the semiconducting polymers. Because pure P3HT is unable to form fibers (due to its low molecular weight), PS (**c**) was added to improve mechanical support for fiber formation.

Electronic-grade p-type regioregular poly(3-hexylthiophene-2,5-diyl) (P3HT) (Mw > 45,000) from Lumtec (New Taipei City, Taiwan) and n-type polymer poly (benzimidazobenzophenanthroline) (BBL) and polystyrene (PS) (Mw = 350,000), both from Sigma-Aldrich (Burlington, MA, USA), (Figure 1), were used as received. Solutions of PS were prepared with anhydrous chloroform (CHCl_3_) and used to dissolve the P3HT (and to allow for subsequent UV/VIS characterization); methanesulfonic acid (MSA), from Sigma-Aldrich (Burlington, MA, USA), was used to dissolve the BBL. Solutions were prepared based on weight percent (wt.%). For the core-material solution of P3HT/PS, 7 wt.% PS/CHCl_3_ was used to lend mechanical support to the P3HT molecules; the final concentration of P3HT in the P3HT/PS/CHCl_3_ blend was 0.4 wt.%. For the shell-material solution, 0.39 wt.% BBL was dissolved in MSA. Each of the two solutions was thoroughly blended with a magnetic stirrer until homogeneous equilibrium was achieved.

PS dissolved in chloroform is colorless; adding P3HT, which is red, imparts a red color to the solution blend. Dissolving BBL in MSA yields a dark red solution; with continued processing with CHCl_3_, BBL changes to a vivid blue-violet color [24]. UV/VIS spectra were obtained (using an Evolution 201 PC spectrometer) for the solutions of pure PS, pure P3HT, blended P3HT/PS, and pure BBL, to obtain the optical distinguishable characteristics of the material solutions.

To form the coaxial nanofibers from these solutions, the electrospinning technique was employed, using two luer-lock syringes and a coaxial needle (Ramé-Hart Instrument Co., Succasunna, NJ, USA). The gauge sizes were 23 G for the core material and 18 G for the shell material. The coaxial needle functioned as an anode, and a sheet of aluminum foil served as a grounded cathode. A programmable syringe pump (pump rate = 3000 µL hr^−1^) was used to maintain a slow, steady flow of the polymeric solutions into the electric field. In this setup, the core needle concentrically separates the polymers that will be extruded into the electric field. Without the application of high voltage, the polymers proceed to meet at the tip and form a pendant drop that quickly reacts by interacting with the solvents and therefore solidifying the polymers. When a voltage is applied to the concentric needles, the electrocapillary effect induces charges on both solutions that change the interfacial tension and consequentially decrease the surface tension, thus enhancing the formation of the charged jet. It is recognized that the high net charge density provided by electroactive polymers in the electrospinning jet increases the induced charges that reduces the surface tension, leading to the formation of nanofibers [32]. At a critical voltage, the formation of the Taylor cone occurs, and the partial solidification of the shell polymer over the core polymer happens while the jet elongates toward the collector, therefore forming the P3HT/PS core and the BBL shell coaxial fibers [33]. This setup resulted in the polymers forming a Taylor cone that emitted a charged jet that continuously moved toward the cathode. Fibers were formed when a critical voltage of 9 kV was applied at the tip of the needle, overcoming the polymer surface tension, stretching the material, enhancing solvent evaporation, and forming the coaxial nanofibers. The formed fibers were collected (i.e., attracted and deposited) onto the aluminum foil and over a silicon wafer (SiO_2_/Si) with prepatterned gold electrodes.

For topological characterization of the nanofibers, TEM grids with 50 µm × 50 µm apertures were passed near the cathode in a weaving motion to collect in-air nanofibers. These fiber samples were first rinsed gently with deionized water to remove any residue of MSA from the surface and then dried for 15 min at 70 °C prior to characterization. Images were taken with a TEM (Phillips FEI Morgagni M 268).

## 3. Results and Discussions

**Figure 2:**
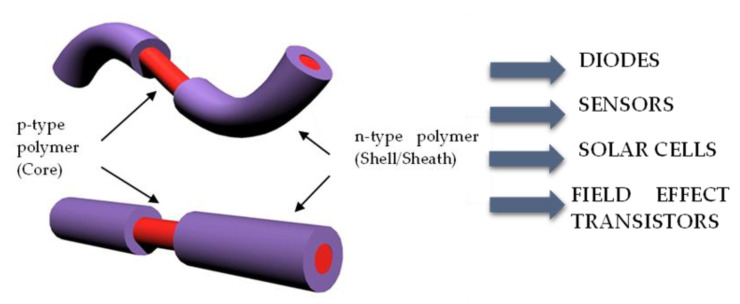
Coaxial nanofiber with p- and n-type semiconducting polymers. This nanofiber provides an opportunity to form p-n junctions that can be tethered for electroactive textiles and single-nanofiber devices.

In this work, coaxial semiconductive polymer-polymer nanofibers were fabricated and morphologically characterized. The homogeneous composite P3HT/PS served as the core, and the BBL solution formed the shell, thereby achieving the desired structure (Figure 2). The p-n junctions thus formed can perform as diodes [34], sensors [35], and transistors [23,36,37,38], as well as photodiodes for solar cell applications [39,40].

**Figure 3:**
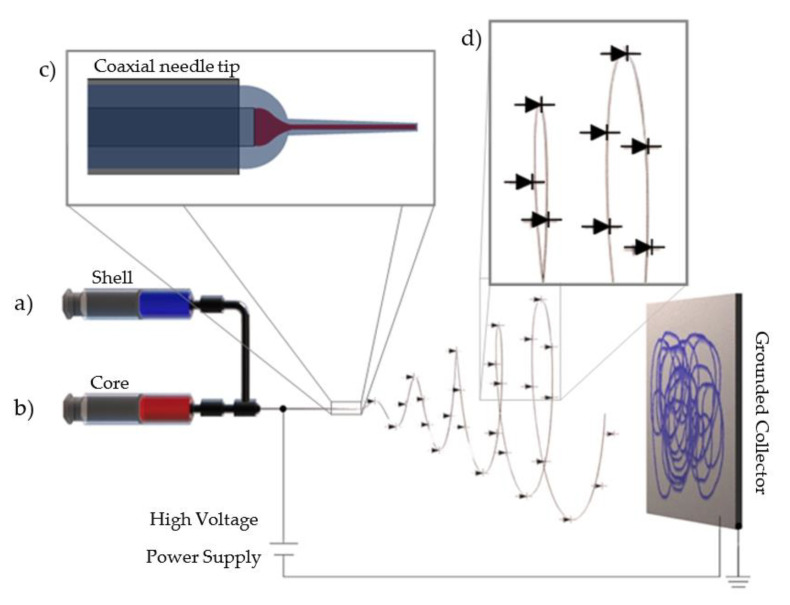
Continuous fabrication of coaxial nanofiber p-n junctions, using the electrospinning technique. The BBL solution (**a**, blue) forms the shell/sheath, and the P3HT/PS solution (**b**, red) forms the core. An electric field overcomes the surface tension, stretching the solution and forming nanofibers. Inset: (**c**) The coaxial needle tip and magnification (**d**) after the Taylor cone formation, with the diode symbols representing the continuous heterojunction formed between the core and the shell.

At low concentrations of P3HT, it is difficult for the electrospinning technique to generate well-formed fibers. This difficulty is due to low molecular weight hindering the formation of molecular entanglements, and this, in turn, results in a solution extensional viscosity too low to form fibers. To overcome this impediment, PS was added to the P3HT solution to provide mechanical support for fiber formation without compromising the semiconductive property of P3HT [41,42,43]. At 7 wt.% PS, the solution was sufficiently viscous and electrically charged by the P3HT that core fibers with a diameter of 200 nm were formed. The BBL solution required no additional polymer for fiber production; the PS in the core-material solution did indirectly help to form and support the final structure. This method could also make the PS fiber composite electroactive as the semiconductor carrier [44]. As noted above, the PS and P3HT polymers were dissolved in CHCl_3_. BBL does not dissolve in CHCl_3_, resulting in the extrusion of uniformly coated BBL shell and P3HT/PS core nanofibers (Figure 3) moving from a viscous to a flexible state, to solvent evaporation and stretching to form a polymer-polymer coaxial nanofiber.

**Figure 4:**
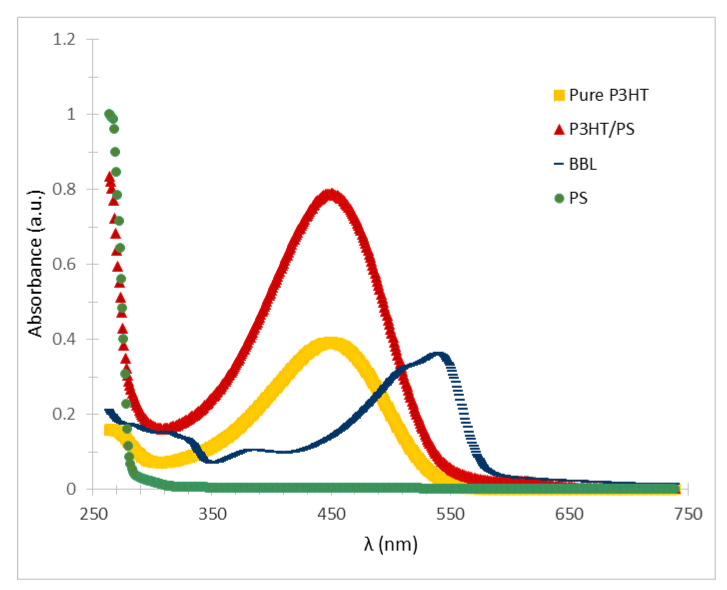
Comparison of the UV/VIS spectra of pure PS (7 wt.%), pure P3HT (2 wt.% in CHCl_3_), and the P3HT/PS (0.4 wt.%/7 wt.%) blend indicates that adding polystyrene does not change the physical or optical characteristics of P3HT. The BBL solution (0.39 wt.%) exhibits its characteristic broad absorption peak at approximately 540 nm.

The UV/VIS spectrum of the P3HT/PS polymer composite was compared to the spectra of the pure (in CHCl_3_) PS and P3HT solution (Figure 4). Over the wavelength range examined, the spectrum of the colorless PS solution is featureless. The P3HT solution exhibits an absorption peak at approximately 450 nm, as does the P3HT/PS blend. This peak is attributable to the π-π* transition of the electronic absorption spectra, as previously documented for pure P3HT [26]. The coincidence of these peak positions indicates that the PS and P3HT polymers are homogeneously integrated within the blend solution, without phase separation or chemical interaction. Measuring the UV/VIS spectra in this way also enabled the BBL to show the absorption peak that defines its optical band gap. The BBL spectrum showed a peak at approximately 380 nm and a wider peak at approximately 540 nm, consistent with previous results [40,45], due to the onset of π-π* transition; this spectrum is characteristic of this ladder polymer [46]. These π-π* transitions determine the band gaps of P3HT and BBL, which are generally 2.2 eV and 1.9 eV, respectively [46,47], in agreement with the optical band gap cutoff values determined from the UV–VIS spectra shown in Figure 4.

**Figure 5:**
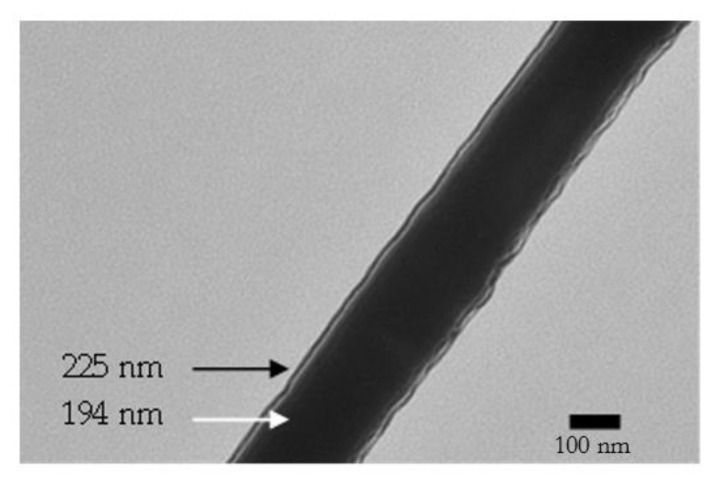
TEM image of a coaxial nanofiber with a P3HT/PS (0.4 wt.%/7 wt.%) core and a BBL (0.39 wt.%) shell. This nanofiber was the smallest one examined. The scale bar is 100 nm.

While the P3HT/PS blend solution showed no evidence of separation or precipitation, the combined solutions of P3HT/PS and BBL were heterogeneous. The reason is that BBL is insoluble in CHCl_3_, and P3HT is insoluble in MSA. These characteristics led to the successful formation of nanometric coaxial fibers. Transmission electron microscopy (TEM) images of the fibers show well-formed core and shell structures (Figure 5). The smallest nanofiber examined had a total diameter of approximately 225 nm, with a core diameter of approximately 194 nm and a stable BBL shell thickness of 31 nm.

**Figure 6:**
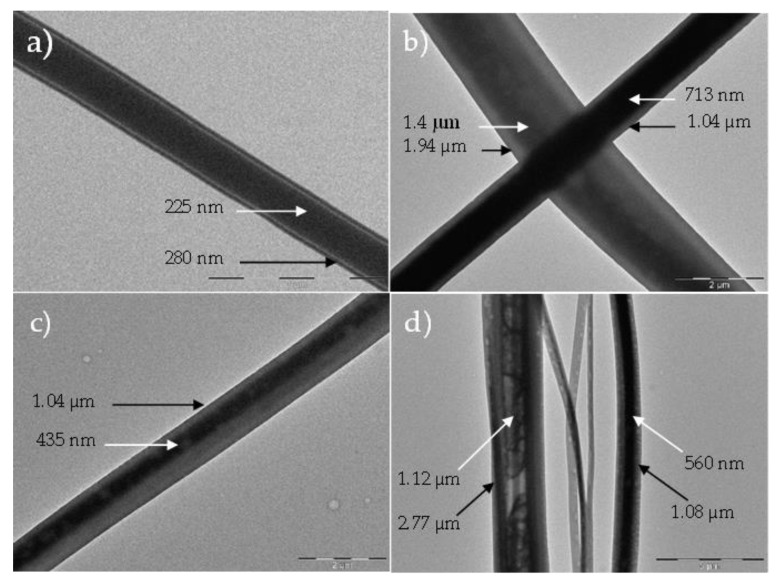
TEM images of coaxial nanofibers (P3HT/PS core (white arrow) with BBL shell (black arrow)) created simultaneously within a single run of the electrospinning technique. Scale bars are 1 µm (**a**), 2 µm (**b**,**c**), and 5 µm (**d**).

With the electrospinning technique, a variety of fiber sizes can be generated within a single production run. However, the diameters of the coaxial nanofiber can be controlled (e.g., solvent-types, concentration of the solutions, molecular weights, and pump rate). Figure 6 shows electrospun fibers from a single run, with outside diameters ranging from approximately 280 (a) to 2772 (d) nanometers. The variety of sizes is due to weak agglomeration at the syringe tip because of quick core-shell interaction, which slightly changes the material extrusion into the electric field. A subsequent production run can yield coaxial nanofibers with a narrow range of diameters. Future work aims to fabricate nanofibers of a single specific diameter by fine-tuning the solution concentrations and feeding rates, controlling the solution’s interaction rate at the concentric needle tip.

**Figure 7:**
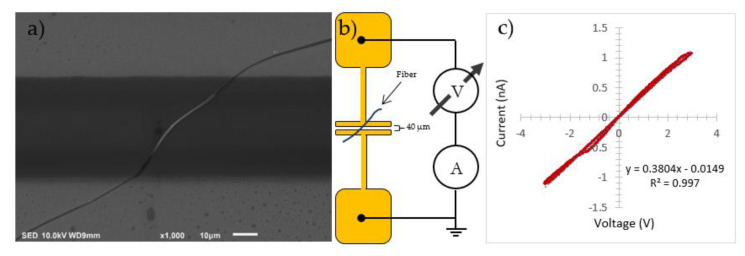
SEM image of coaxial nanofiber electrically connected to the BBL shell (**a**), device structure with electrode separation of 40 µm (**b**), and current-voltage (I–V) characterization of a single fiber (**c**).

Electrical characterization was performed for a single nanofiber, collected between two gold electrodes during the electrospinning process, as shown in Figure 7. The fiber was deposited by rapidly waving the electrodes perpendicularly to the spinning direction near the collector. The formed device was dipped in deionized water and vacuum dried at 70 °C for 15 min, ensuring electrical contact on the electrodes. The fiber has a diameter of 1.2 µm with electrode separation of 40 µm. The electrical connections are made directly to the shell of the fiber, therefore characterizing the BBL. The fiber showed a linear IV characterization with a conductivity of ~ 1.4×10^−4^ S/m.

## 4. Conclusions

In conclusion, in this paper, we described, for the first time, the electrospinning fabrication of pure-polymer composite coaxial P3HT/PS-BBL fibers to form a p-n junction coaxial structure. The electrically active materials used in this work are the electronic-grade regioregular P3HT and the ladder polymer BBL, with PS providing mechanical support for the thin fiber P3HT core. Our UV–VIS analysis shows that no chemical or physical changes occurred when the P3HT was blended with the PS. Strategic solvent selection ensures the formation of electrospun fibers that are simultaneously coated with a protective BBL shell. TEM imaging characterizations show that the formed composite fibers had diameters ranging from approximately 280 to 2.77 µm. We expect this coaxial structure not only to support the development of flexible, multifunctional electronic devices, including tunable diodes for UV radiation detectors and rectifiers for integrated intelligent textiles, but also to be able to sense, communicate, and generate its own energy, all in a single nanofiber. The ability to use the electrospinning technique to make electroactive coaxial nanofibers from novel organic polymer-based structures will have profound implications for the construction of the mentioned electronic devices.

## 5. Patents

Patent number 10,629,814—Coaxial Semiconductive Organic Nanofibers and Electrospinning Fabrication.

## Data Availability

Not applicable.

## References

[1] Shi S., Si Y., Han Y., Wu T., Iqbal M.I., Fei B., Li R.K.Y., Hu J., Qu J. (2021). Recent Progress in Protective Membranes Fabricated via Electrospinning: Advanced Materials, Biomimetic Structures, and Functional Applications. Adv. Mater..

[2] Park K., Kang S., Park J., Hwang J.J. (2021). Fabrication of silver nanowire coated fibrous air filter medium via a two-step process of electrospinning and electrospray for anti-bioaerosol treatment. Haz. Mat..

[3] Yang L., Zhao Y., Cui D., Liu Y., Zou Q., Xu S., Luo S., Ye C. (2022). Coaxial bioelectrospinning of P34HB/PVA microfibers biomimetic scaffolds with simultaneity cell-laden for improving bone regeneration. Mat. Des..

[4] Han D., Steckl A.J. (2019). Coaxial electrospinning formation of complex polymer fibers and their applications. Chem. Plus Chem..

[5] Li J., Liu Y., Abdelhakim H.E. (2022). Drug Delivery Applications of Coaxial Electrospun Nanofibres in Cancer Therapy. Molecules.

[6] Serrano-Garcia W., Jayathilaka W.A.D.M., Chinnappan A., Tran T.Q., Baskar C., Thomas S., Ramakrishna S. (2019). Nanocomposites for electronic applications that can be embedded for textiles and wearables. Sci. China. Tech. Sci..

[7] Jayathilaka W.A.D.M., Qi K., Qin Y., Chinnappan A., Serrano-Garcia W., Chinnappan B., Wang H., He J., Cui S., Thomas S. (2019). Significance of nanomaterials in wearables: A review on wearable actuators and sensors. Adv. Mater..

[8] Bayan M.A.H., Taromi F.A., Lanzi M., Pierini F. (2021). Enhanced efficiency in hollow core electrospun nanofiber-based organic solar cells. Sci. Rep..

[9] Ma W., Yang C., Gong X., Lee K., Heeger A.J. (2005). Thermally stable, efficient polymer solar cells with nanoscale control of the interpenetrating network morphology. Adv. Funct. Mater..

[10] Fukuda K., Takeda Y., Mizukami M., Kumaki D., Tokito S. (2014). Fully solution-processed flexible organic thin film transistor arrays with high mobility and exceptional uniformity. Sci. Rep..

[11] Luzio A., Canesi E.V., Bertarelli C., Caironi M. (2014). Electrospun polymer fibers for electronic applications. Materials.

[12] Kleinschmidt A.T., Root S.E., Lipomi D.J. (2017). Poly(3-hexylthiophene) (P3HT): Fruit fly or outlier in organic solar cell research?. Mater. Chem..

[13] Chang H.C., Liu C.L., Chen W.C. (2013). Flexible nonvolatile transistor memory devices based on One-Dimensional electrospun P3HT: Au hybrid nanofibers. Adv. Funct. Mater..

[14] Irimia-Vladu M. (2014). “Green” electronics: Biodegradable and biocompatible materials and devices for sustainable future. Chem. Soc. Rev..

[15] Jin G., Prabhakaran M.P., Kai D., Kotaki M., Ramakrishna S. (2012). Electrospun photosensitive nanofibers: Potential for photocurrent therapy in skin regeneration. Photochem. Photobiol. Sci..

[16] Guarino V., Altobelli R., Cirillo V., Cummaro A., Ambrosio L. (2015). Additive electrospraying: A route to process electrospun scaffolds for controlled molecular release. Polym. Adv. Technol..

[17] Moge A.K., Gupta B.S. (2008). Co-axial electrospinning for nanofiber structures: Preparation and applications. Polym. Rev..

[18] Li Q., Ding S., Zhu W., Feng L., Dong H., Hu W.J. (2016). Recent advances in one-dimensional organic p-n heterojunctions for optoelectronic device applications. Mater. Chem. C.

[19] Aleshin A.N. (2006). Polymer Nanofibers and Nanotubes: Charge Transport and Device Applications. Adv. Mater..

[20] Gonzales R., Pinto N.J. (2005). Electrospun poly (3-hexylthiophene-2, 5-diyl) fiber field effect transistor. Synth. Met..

[21] Chang J.F., Sun B., Breiby D.W., Nielsen M.M., Sölling T.I., Giles M., McCulloch I., Sirringhaus H. (2004). Enhanced mobility of poly (3-hexylthiophene) transistors by spin-coating from high-boiling-point solvents. Chem. Mater..

[22] Merlo J.A., Frisbie C.D.J. (2004). Field effect transport and trapping in regioregular polythiophene nanofibers. Phys. Chem. B.

[23] Babel A., Jenekhe S.A. (2003). High electron mobility in ladder polymer field-effect transistors. JACS.

[24] Briseno A.L., Mansfeld S.C.B., Shamberger P.J., Ohuchi F.S., Bao Z., Jenekhe S.A., Xia Y. (2008). Self-assembly, molecular packing, and electron transport in n-type polymer semiconductor nanobelts. Chem. Mater..

[25] Serrano W., Pinto N.J. (2012). Electrospun fibers of poly (vinylidene fluoride-trifluoroethylene)/poly (3-hexylthiophene) blends from tetrahydrofuran. Ferroelectrics.

[26] Serrano W., Meléndez A., Ramos I., Pinto N.J. (2016). Poly (lactic acid)/poly (3-hexylthiophene) composite nanofiber fabrication for electronic applications. Polym. Int..

[27] Yoo H.S., Kim T.G., Park T.G. (2009). Surface-functionalized electrospun nanofibers for tissue engineering and drug delivery. Adv. Drug Deliv. Rev..

[28] Reese M.O., Morfa A.J., White M.S., Kopidakis N., Shaheen S.E., Rumbles G., Ginley D.S. (2008). Pathways for the degradation of organic photovoltaic P3HT:PCBM based devices. Solar Energy Mater. Solar Cells.

[29] Briseno A.L., Kim F.S., Babel A., Xia Y., Jenekhe S.A. (2011). n-Channel polymer thin film transistors with long-term air-stability and durability and their use in complementary inverters. J. Mater. Chem..

[30] Anthony J.E. (2014). Addressing challenges. Nat. Mater..

[31] Serrano-Garcia W. (2021). Advanced Organic Polymers for the Nanoscale Fabrication of Fiber-Based Electronics Using the Electrospinning Technique. USF Tampa Graduate Theses and Dissertations. https://digitalcommons.usf.edu/etd/9228.

[32] Sundarrajan S., Murugan R., Nair A.S., Ramakrishna S. (2010). Fabrication of P3HT/PCBM solar cloth by electrospinning technique. Mater. Lett..

[33] Rathore P., Schiffman J.D. (2021). Beyond the single-nozzle: Coaxial electrospinning enables innovative nanofiber chemistries, geometries, and applications. ACS Appl. Mater. Interfaces.

[34] Pinto N.J., Carrasquillo K.V., Rodd C.M., Agarwal R. (2009). Rectifying junctions of tin oxide and poly (3-hexylthiophene) nanofibers fabricated via electrospinning. Appl. Phys. Lett..

[35] Gao Q., Meguro H., Okamoto S., Kimura M. (2012). Flexible tactile sensor using the reversible deformation of poly (3-hexylthiophene) nanofiber assemblies. Langmuir.

[36] Lee S., Moon G.D., Jeong U.J. (2009). Continuous production of uniform poly (3-hexylthiophene)(P3HT) nanofibers by electrospinning and their electrical properties. Mater. Chem..

[37] Lee S.W., Lee H.J., Choi J.H., Koh W.G., Myoung J.M., Hur J.H., Park J.J., Cho J.H., Jeong U. (2010). Periodic array of polyelectrolyte-gated organic transistors from electrospun poly (3-hexylthiophene) nanofibers. Nano. Lett..

[38] Babel A., Wind J.D., Jenekhe S.A. (2004). Ambipolar charge transport in air-stable polymer blend thin-film transistors. Adv. Funct. Mater..

[39] Jenekhe S.A., Yi S. (2000). Efficient photovoltaic cells from semiconducting polymer heterojunctions. Appl. Phys. Lett..

[40] Alam M.M., Jenekhe S.A. (2004). Efficient solar cells from layered nanostructures of donor and acceptor conjugated polymers. Chem. Mater..

[41] Qiu L., Wang X., Lee W.H., Lim J.A., Kim J.S., Kwak D., Cho K. (2009). Organic thin-film transistors based on blends of poly (3-hexylthiophene) and polystyrene with a solubility-induced low percolation threshold. Chem. Mater..

[42] Qiu L., Lim J.A., Wang X., Lee W.H., Hwang M., Cho K. (2008). Versatile use of vertical-phase-separation-induced bilayer structures in organic thin-film transistors. Adv. Mater..

[43] Hur J., Cha S.N., Im K., Lee S.W., Jeong U., Kim J., Park J.J. P3HT-PS blend nanofiber FET based on electrospinning. Proceedings of the 10th International Conference on Nanotechnology: Joint Symposium with NANO Korea 2010.

[44] Serrano W., Meléndez A., Ramos I., Pinto N.J. (2014). Electrospun composite poly (lactic acid)/polyaniline nanofibers from low concentrations in CHCl3: Making a biocompatible polyester electro-active. Polymer.

[45] Jenekhe S.A., de Paor L.R., Chen X.L., Tarkka R.M. (1996). Photoinduced electron transfer in binary blends of conjugated polymers. Chem. Mater..

[46] Sun Q., Park K., Dai L.J. (2009). Liquid crystalline polymers for efficient bilayer-bulk-heterojunction solar cells. Phys. Chem. C.

[47] Narayan K.S., Taylor-Hamilton B.E., Spry R.J., Ferguson J.B. (1995). Photoconducting properties of a ladder polymer. J. Appl. Phys..

